# Disease-Modifying Effects of Long-Term and Continuous Use of Nonsteroidal Anti-Inflammatory Drugs (NSAIDs) in Spondyloarthritis

**DOI:** 10.1155/2019/5324170

**Published:** 2019-01-29

**Authors:** Rebecca S. Y. Wong

**Affiliations:** Faculty of Medicine, SEGi University, No. 9, Jalan Teknologi, Taman Sains Selangor, Kota Damansara, PJU 5, 47810 Petaling Jaya, Selangor, Malaysia

## Abstract

Spondyloarthritis or spondyloarthropathy (SpA) is a group of related rheumatic disorders, which presents with axial and nonaxial features, affecting structures within the musculoskeletal system, as well as other bodily systems. Both pharmacological and nonpharmacological therapeutic options are available for SpA. For decades, nonsteroidal anti-inflammatory drugs (NSAIDs) have been used as the first-line drugs to treat the disease. Research has shown that other than pain relief, NSAIDs have disease-modifying effects in SpA. However, to achieve these effects, continuous and/or long-term NSAID use is usually required. This review will give an overview of SpA, discuss NSAIDs and their disease-modifying effects in SpA, and highlight some of the important adverse effects of long-term and continuous NSAID use, particularly those related to the gastrointestinal, renal, and cardiovascular systems.

## 1. Introduction

Spondyloarthritis or spondyloarthropathy (SpA) includes a family of inflammatory diseases commonly affecting the joints, bones, ligaments, and tendons. These diseases are related in many ways as they have several similarities in their genetic and clinical features. SpA is potentially severe and disabling, and may lead to a reduced lifespan [1]. Patients with SpA may experience chronic pain in axial and peripheral joints, affecting their normal functioning and quality of life. Therefore, the main goal in the management of SpA is to reduce disease activity and improve the quality of life. Many drugs have been used to treat SpA such as NSAIDs, disease-modifying anti-inflammatory drugs (DMARDs), corticosteroids, and biologic drugs [2–5].

NSAIDs are generally the first-line drugs used in the treatment of SpA [2]. Other than their analgesic effects, research has shown that NSAIDs exhibit disease-modifying effects in SpA [6]. However, for these changes to take place, the patients are usually required to take NSAIDs on a continuous and/or long-term basis. There are many pros and cons of using NSAIDs. For instance, NSAIDs have the advantage of price over the biologics as the latter are normally much more expensive than the former. However, continuous long-term use of NSAIDs is not without disadvantages. This article gives an overview of spondyloarthritis, NSAIDs, and their disease-modifying effects in SpA, as well as the accompanying adverse effects of these drugs.

## 2. Spondyloarthritis

SpA encompasses a group of interrelated inflammatory diseases including ankylosing spondylitis (AS), psoriatic arthritis, arthritis related to inflammatory bowel disease (or enteropathic arthritis), and reactive arthritis, as well as undifferentiated SpA [7]. As a whole, patients with SpA can be broadly divided into two main groups, i.e., those with axial SpA and those with peripheral SpA only [8]. Patients in the first group are characterized by sacroiliitis on imaging or positive human leukocyte antigen- (HLA-) B27. On the other hand, those with peripheral manifestations only are characterized by peripheral arthritis, enthesitis, or dactylitis. Both groups of patients also present with other SpA features specified in the Assessment of SpondyloArthritis International Society (ASAS) classification criteria [8].

HLA-B27 is one of the most important genetic factors in the pathogenesis of SpA. Individuals who are HLA-B27 positive are at increased risk of SpA. In a French study that investigated the prevalence of SpA in reference to HLA-B27, it was reported that 75% of patients with SpA were HLA-B27 positive as compared to 6.9% among healthy controls [9]. Besides increasing the risk of SpA, HLA-B27 positivity has been linked to disease presentation. For instance, HLA-B27-positive individuals were reported to have an earlier onset of AS compared to those who were HLA-B27 negative [10]. In one study, HLA-B27 was associated with the severity (*p*=0.009) and number of sacroiliac (SI) joint lesions (*p*=0.045), as well as the persistence of inflammation at one year (*p*=0.02) [11].

As a result of the differences in ethnicity and geographical and genetic backgrounds, as well as the application of different diagnostic criteria, the prevalence of SpA varies in different studies and populations. For example, the pooled prevalence of SpA has a range of 0.20% (Southeast Asia) to 1.61% (Northern Arctic communities) [12]. In contrast to rheumatoid arthritis, SpA has an early onset, usually before the age of 45 years, affecting males more frequently than females. Inflammatory back pain remains one of the most characteristic features of the disease. Other clinical features of SpA include peripheral arthritis, dactylitis, enthesitis, eye involvement (uveitis), and neurological manifestations secondary to spinal fractures and abnormalities [13].

Both nonpharmacologic and pharmacologic treatment options are available for treating patients with SpA. For nonpharmacologic management, patient education and support groups have shown to be beneficial, whereas physical therapy and exercise have been reported to be useful in symptom control [2, 14]. As for pharmacologic treatment, NSAIDs are to be used as the first-line drugs to treat the disease for patients with axial SpA (both radiographic and nonradiographic), according to the recommendations set by the ASAS/European League Against Rheumatism (EULAR) (update 2016), with important consideration given to the potential side effects of the drugs. Hence, they should be prescribed only if the patients are symptomatic [2]. DMARDs such as sulphasalazine and methotrexate have also been reported to play a role in the pharmacologic treatment of SpA [2, 3]. Although DMARDs are effective in treating peripheral SpA [5], they are generally not very useful in patients with axial involvement [15] while corticosteroids are only used locally at sites of inflammation, and they are usually not recommended for axial disease [2]. On the other hand, systemic steroids are rarely used in SpA. However, short-term systemic steroids have been shown to benefit some patients who have active disease despite taking NSAIDs [4].

In the past two decades, there have been many new developments in the treatment of SpA, such as the use of biologic drugs in severe cases (reviewed by Bruner et al.) [16]. The use of these biological agents (e.g., tumour necrosis factor- (TNF-) alpha blockers like infliximab, adalimumab, and etanercept) has brought revolutionary changes in the management of the disease. However, the use of biologics is often accompanied by serious side effects such as life-threatening infections [17]. Some researchers have also explored the role of stem cell-based therapies in SpA (reviewed by Wong) [18]. Nevertheless, more research and exploration are necessary before stem cell-based therapies are used widely in the treatment of SpA.

Potential candidates for TNF-alpha blocker treatment have to fulfil several criteria according to the ASAS/EULAR recommendations [2]. The patient must have radiographic evidence of sacroiliitis, either an increased C-reactive protein (CRP) or magnetic resonance imaging (MRI) evidence of active sacroiliitis and a high disease activity as measured by the Ankylosing Spondylitis Disease Activity Score (ASDAS) and Bath Ankylosing Spondylitis Disease Activity Index (BASDAI), despite the use of at least two different NSAIDs at their maximal dose, for at least 4 weeks in total. High disease activity is defined as an ASDAS ≥2.1 or BASDAI ≥4.0. For patients with predominant peripheral manifestations, failure of a local steroid injection (if appropriate) or a therapeutic trial of sulphasalazine are normally required. It is worth mentioning that not all biologics are suitable in treating the disease, as the ASAS/EULAR recommendations only suggest two classes of biological DMARDs, i.e., TNF-alpha blockers and the interleukin (IL) 17 inhibitor secukinumab for the treatment of SpA.

Interestingly, the response of treatment to TNF-alpha blockers is genetically linked to HLA-B27. In a systemic review and meta-analysis, Maneiro et al. explored the predictors of response to TNF-alpha blocker treatment. In all the articles analysed, 6737 patients with AS were included in 37 articles and 4034 patients with psoriatic arthritis were included in 23 articles. It was reported that the genetic factor HLA-B27 was a predictor of a better response to TNF-alpha blockers. Other factors that were reported to be predictors of a better response to these drugs include young age, male sex, low-baseline Bath Ankylosing Spondylitis Functional Index (BASFI), high-baseline BASDAI, and CRP in patients with AS. However, no predictors were identified for patients with psoriatic arthritis [19]. On the other hand, the literature on the relation between genetic factors (such as HLA-B27) and the response to NSAIDs is scarce. However, in one study that explored genetic variability in cyclooxygenase (COX) enzyme regulation, it was reported that there was no significant difference in the genotype of COX polymorphism with respect to the response to NSAIDs in patients with axial SpA [20].

## 3. Continuous Long-Term Use of NSAIDs and Their Disease-Modifying Effects in SpA

NSAIDs are drugs commonly used in the clinical practice and have shown to be effective over placebo in both acute and chronic pain [21]. Due to their analgesic and anti-inflammatory effects, NSAIDS are frequently used in many rheumatic disorders [22] such as rheumatoid arthritis and SpA. These drugs reduce pain and inflammation by blocking the enzyme cyclooxygenase (COX). There are two isoforms of COX, i.e., COX-1 and COX-2. The former has a homeostatic and housekeeping role and is expressed constitutively and widely in cells and tissues while the latter is responsible for the generation of prostaglandins in fever, inflammation, and carcinogenesis [23].

One way to classify NSAIDs is based on their selectivity on COX-1 and COX-2 [24, 25]. In general, NSAIDs can be classified into (a) nonselective COX inhibitors (e.g., ibuprofen, naproxen, ketoprofen, and indomethacin), (b) preferential COX-2 inhibitors (e.g., nimesulide and meloxicam), and (c) selective COX-2 inhibitors (e.g., celexocib, etoricoxib, and rofecoxib) [26]. It is worth mentioning that, on 30 September 2004, rofecoxib was voluntarily withdrawn worldwide by Merck and Co, as it has been shown that long-term use of the drug leads to twofold increased risk in myocardial infarction in comparison with placebo [27].

In SpA, despite many years of NSAID use as a first-line pharmacologic treatment, many questions still remain unanswered. Such questions include whether or not NSAIDs should be used on a long-term and continuous basis in patients with SpA and whether or not NSAIDs exhibit disease-modifying effects on SpA. Thus far, findings from previous studies are contradictory. In one study by Sieper et al., patients with AS were divided into two groups, one group receiving continuous diclofenac and the second, on-demand diclofenac. At the end of two years, there was no significant reduction in radiographic progression in the former compared with the latter [28].

However, several studies have shown that other than pain relief, NSAIDs exhibit disease-modifying effects in SpA, which can be dated back to the 1970s. Boersma demonstrated in a retrospective study with 40 patients of AS that continuous phenylbutazone retarded or delayed ossification of the lumbar vertebral column. On the other hand, the control group showed rapid progression of ossification [29]. In 2005, a study by Wanders et al. showed that a significant difference in the modified Stoke Ankylosing Spondylitis Spine Score (mSASSS) was observed between AS patients who were on continuous NSAIDs (celecoxib or ketoprofen) and those taking NSAIDs on demand. The first group had a mean score of 0.4 ± 1.7, while the latter had a mean score of 1.5 ± 2.5 (*p*=0.002). Although the first group of patients had a higher frequency of adverse events, the difference was statistically not significant, suggesting that continuous NSAIDs play a role in reducing radiographic progression without increasing toxicity [6].

In a two-year study, Poddubnyy et al. reported an association between a high NSAID intake (an NSAID dose equivalent of 150 mg of diclofenac daily) and a lower likelihood of significant radiographic progression in AS patients (mSASSS progression = 0.14 ± 1.80) when compared to patients who were on a low NSAID index (mSASSS progression = 4.36 ± 4.53; *p*=0.045). In the study, an NSAID index >50 was considered as a high NSAID intake. The disease-modifying effects of NSAIDs were most obvious in those with baseline syndesmophytes and an increased CRP [30].

On the other hand, in a six-week study by Varkas et al., it was shown that NSAIDs significantly reduced bone marrow oedema. In the study, an optimal dose of NSAIDs was given to 30 patients with axial SpA and positive SI joint findings on MRI. One-third of the patients dropped out of the study due to intolerance of the full dose of NSAIDs. Of those who continued to the end, a significant decrease in signal intensity of SI joint bone marrow oedema (*p*=0.001) was observed at week 6 compared to baseline [31].

## 4. Adverse Effects of NSAIDs

Thus far, the studies that investigated the disease-modifying effects of NSAIDs in SpA required long-term, continuous use of NSAIDs. The duration ranged from 6 weeks to 2 years. However, long-term NSAID therapy is not without side effects and complications. There are numerous studies in this area of research, and the data available in the published literature are overwhelming. This section will highlight the main adverse effects of NSAIDs particularly the gastrointestinal, renal, and cardiovascular adverse effects.

### 4.1. Gastrointestinal Adverse Effects

Gastrointestinal (GI) adverse effects are common among NSAID users. These may range from minor symptoms like dyspepsia, nausea, and heartburn to severe, life-threatening gastrointestinal bleed. The risk of uncomplicated and complicated GI adverse effects increases in older patients and those taking other drugs (e.g., aspirin and corticosteroids) concomitantly [32]. An earlier study reported that among patients with osteoarthritis or rheumatoid arthritis treated with NSAIDs, 24% showed peptic ulcers on endoscopy [33]. The decline in GI adverse effects in NSAID treatment is attributed to dose reduction, the use of proton pump inhibitors, and the use of COX-2 selective NSAIDs [34].

Past research has shown that COX-2 inhibitors reduce GI toxicity and complications when compared to conventional NSAIDs [35]. While some believe that selective COX-2 inhibitors cause less GI toxicity and side effects [36], others do not agree that they are more superior. In 2002, Budenholzer argued that even though the GI adverse events are less frequent with rofecoxib, it is less safe than naproxen [37]. This was shortly followed by the global withdrawal of rofecoxib in 2004 [27]. Although earlier research suggests that NSAID-related gastric injury is mainly due to COX-1 inhibition [38], it is currently believed that both COX-1 and COX-2 play a role in the maintenance of gastric mucosal integrity [39].

### 4.2. Renal Adverse Effects

Approximately 1–5% of NSAID users develop renal adverse effects [40]. Various renal side effects of NSAIDs have been reported with NSAID use. These include a reduction in glomerular filtration rate (GFR), acute renal failure, renal papillary necrosis, nephrotic syndrome, acute interstitial nephritis (AIN), and chronic renal failure, as well as fluid and electrolyte retention (reviewed by Harirforoosh et al.) [41]. In an early study, Whelton et al. demonstrated that patients on naproxen (−5.31 mL/min per 1.73 m^2^) showed a significantly greater decrease in GFR compared to those on celecoxib (-0.86 mL/min per 1.73 m^2^) on day 6 of the treatment (*p*=0.004) [42]. However, another study showed COX-2 selective (celecoxib) and nonselective NSAIDs (indomethacin) showed similar renal effects in elderly patients [43].

In another study that investigated the risk factors of NSAID-induced acute kidney injury (AKI) among patients with hyperuricaemia, it was shown that the mean age of those developing AKI was significantly higher (*p*=0.008), while the baseline GFR (*p*=0.001), serum albumin level (*p* < 0.0001), and serum haemoglobin level (*p* < 0.0001) were significantly lower in this group of patients. However, there were no statistically significant differences between AKI and non-AKI group of patients in terms of gender, body mass index (BMI), NSAID selectivity, and the presence of diabetes mellitus or hypertension (*p* > 0.05) [44].

NSAID-induced AKI is believed to be due to two different mechanisms. In the first mechanism, AKI is due to a reduction of prostaglandins, which leads to a decrease in renal plasma flow. In AKI, there is interruption in the compensatory vasodilation response of prostaglandins to the vasoconstriction induced by the body's hormones [45]. The second mechanism is due to AIN. Inflammatory cell infiltrates are characteristically found in the interstitium of patients with AIN due to an immunological reaction in response to NSAID exposure [46].

### 4.3. Cardiovascular Adverse Effects

One of the cardiovascular adverse effects of NSAIDs is that these drugs worsen hypertension. They are believed to worsen hypertension by (a) inhibition of antihypertensive drugs (e.g., angiotensin-converting enzyme (ACE) inhibitors and angiotensin receptor blockers) [47], (b) activation of the renin-angiotensin-aldosterone system as a result of NSAID-induced acute renal failure, or (c) aggravation of preexisting renal dysfunction [48]. Other possible mechanisms in NSAID-induced hypertension include salt and water retention as a result of reduced renal arterial production of prostaglandin or an increase in peripheral vascular resistance secondary to stimulation of endothelin-1 synthesis and inhibition of prostaglandin synthesis [49].

NSAID use has also been linked to an increased risk in myocardial infarction (MI). In one Finnish population-based matched case-control study, an adjusted odds ratio of 1.40 (95% CI, 1.33–1.48) was reported for the risk of first MI with current use of NSAIDs. The study also demonstrated that regardless of NSAID selectivity, the risk was found to be similar among different NSAIDs and that the cardiovascular risk was not consistently modified by age. In addition, none of the NSAIDs showed any MI-protective effects. The study, therefore, concluded that conventional and COX-2 selective NSAIDs were associated with an increased risk of MI [50].

## 5. Conclusions

Several important points can be made from this review. Firstly, there are not many studies in the published literature on the disease-modifying effects of NSAIDs in SpA, and the sample size in these studies is usually small. Secondly, findings on the disease-modifying effects of NSAIDs are contradictory with some showing no significant effects while others showing a retardation or delay in radiographic progression of disease or reduction in bone marrow oedema. Thirdly, different types of NSAIDs were used in each study, making comparison of the disease-modifying effects in SpA challenging. Lastly, there are many side effects associated with long-term use of NSAIDs. In view of the limited data and the numerous potentially serious adverse effects of NSAIDs, it is recommended that more large-scale studies are necessary to support the disease-modifying effects of continuous, long-term NSAID therapy in SpA. Until then, NSAIDs should be used with great care and only when the benefits outweigh the risks in SpA.

## Abbreviations

ACE: Angiotensin-converting enzyme
AIN: Acute interstitial nephritis
AKI: Acute kidney injury
AS: Ankylosing spondylitis
ASAS: Assessment of SpondyloArthritis International Society
ASDAS: Ankylosing Spondylitis Disease Activity Score
BASDAI: Bath Ankylosing Spondylitis Disease Activity Index
BASFI: Bath Ankylosing Spondylitis Functional Index
BMI: Body mass index
COX: Cyclooxygenase
CRP: C-reactive protein
DMARDs: Disease-modifying antirheumatic drugs
EULAR: European League Against Rheumatism
GFR: Glomerular filtration rate
GI: Gastrointestinal
HLA: Human leukocyte antigen
IL: Interleukin
MI: Myocardial infarction
MRI: Magnetic resonance imaging
mSASSS: modified Stoke Ankylosing Spondylitis Spine Score
NSAIDs: Nonsteroidal anti-inflammatory drugs
SI: Sacroiliac
SpA: Spondyloarthropathy or spondyloarthritis
TNF: Tumour necrosis factor.
